# Revealing the psychopathological pathway linking trauma to post-traumatic stress disorder: longitudinal network approach

**DOI:** 10.1192/bjo.2023.615

**Published:** 2023-12-04

**Authors:** Chen Chen, Chengqi Cao, Ruojiao Fang, Li Wang, Denny Borsboom

**Affiliations:** Laboratory for Traumatic Stress Studies, CAS Key Laboratory of Mental Health, Institute of Psychology, Chinese Academy of Sciences, China; and Department of Psychology, University of Chinese Academy of Sciences, China; Department of Psychological Methods, University of Amsterdam, The Netherlands

**Keywords:** Post-traumatic stress disorder, network approach, directed acyclic graph, longitudinal, child

## Abstract

The present study investigated the psychopathological processes of post-traumatic stress disorder (PTSD) following the network approach to psychopathology. The directed acyclic graph model was employed to analyse a large longitudinal data-set of Chinese children and adolescents exposed to a destructive earthquake. It was found that intrusion symptoms were first activated by trauma exposure, and subsequently activated other PTSD symptoms. The data are consistent with the idea that symptoms may form a self-sustaining dynamic network by interacting with each other to promote or maintain the chronicity of PTSD. The findings advance the current understanding about the psychopathological processes of PTSD, and inform further research and clinical practices on post-traumatic psychopathology.

Post-traumatic stress disorder (PTSD) is a severe and debilitating mental disorder precipitated by exposure to potentially traumatic stressors. Although various psychopathological explanations of PTSD have been proposed by different theoretical models,^1^ how trauma exposure, the nomological prerequisite for PTSD, is linked to the psychopathological processes of PTSD remains unclear. Three hypotheses regarding the role of trauma exposure in triggering PTSD have been proposed: (a) as a common cause of PTSD symptoms; (b) as a trigger that activates specific gateway symptoms and (c) as a chronic stressor that not only ignites onset, but also serves the maintenance of PTSD.^2^ The network approach to psychopathology offers a promising way to test these hypotheses comprehensively. This approach characterises mental disorders as networks of psychopathological symptoms that causally interact, and mental disorders arise from external trigger events activating certain symptoms, which subsequently activate other symptoms of the networks.^3^ During the past several years, the network approach has been widely used to investigated PTSD.^4^ However, these studies were largely limited by mainly focusing on PTSD symptoms themselves, and using cross-sectional data and statistical models such as the Gaussian graphical model (GGM), which can only yield evidence for undirected relationships rather than potential causal relationships. To address the research gap and further advance extant knowledge on PTSD psychopathology, this study aimed to employ the directed acyclic graph (DAG; a commonly used model in the causal inference literature^5^) to analyse a large longitudinal data-set of Chinese children and adolescents exposed to a destructive earthquake. Two research questions were investigated, including how trauma exposure links to the psychopathological processes of PTSD, and how PTSD symptoms dynamically interact with each other in the natural course of PTSD.

## Method

The sample comprised 4910 children and adolescents (49.5% boys) who experienced the 2008 Wenchuan earthquake, with a mean age of 11.4 (s.d. = 1.4) years at the first survey. Four surveys were conducted at 2.5 (time point 1), 3.5 (time point 2), 4.5 (time point 3) and 5.5 (time point 4) years after the earthquake. The research protocol was approved by the Institutional Review Board of Institute of Psychology, Chinese Academy of Sciences (approval number H11021), and written consent was obtained from students and their guardians.

Trauma exposure was assessed with five dichotomous questions about earthquake-related traumatic experiences (for item contents, see Supplementary material available at https://doi.org/10.1192/bjo.2023.615) at time point 1, and a total score was calculated by summing question responses. PTSD symptoms were measured with the University of California, Los Angeles PTSD Reaction Index for DSM-IV.^6^ As including too many nodes in a network model could compromise the validity of causal inferences,^5^ we used the scores of PTSD symptom clusters rather than single symptoms in the subsequent analyses. Based on the well-validated five-factor dysphoric arousal model of DSM-IV PTSD symptoms,^7^ the scores of five symptom clusters (intrusion, avoidance, numbing, dysphoric arousal and anxious arousal) were calculated by summing the responses on items corresponding to each cluster.

A DAG was first estimated with the iamb algorithm in the R package bnlearn (version 4.0.3 for Windows; see http://www.R-project.org/).^8^ As future symptoms could not influence past events, edge directions in the network were forced to respect time order by blocking all edges projecting back in time. To evaluate the different roles of symptom clusters in psychopathological processes of PTSD, we represented symptom clusters as nodes in a network structure, and assessed each node's centrality in the network to determine how symptom clusters were connected to each other within each measurement. Three centrality indices of nodes were computed, including outdegree, indegree and degree (i.e. number of edges departing from, arriving at and connecting to a node, respectively). The robustness of DAG and stability of centrality were evaluated through bootstrapping (1000 replicates).

Additional GGMs were further performed with the R package qgraph,^9^ to rule out alternative explanations of the observed relations between trauma exposure and PTSD symptoms. Partial correlation networks of PTSD symptom clusters were estimated including and not including trauma exposure, to test whether trauma exposure acts as a common cause of PTSD symptoms. If trauma exposure operated as a common cause, connections among symptom clusters would vanish when trauma exposure was included in the model.

## Results

**Fig. 1:**
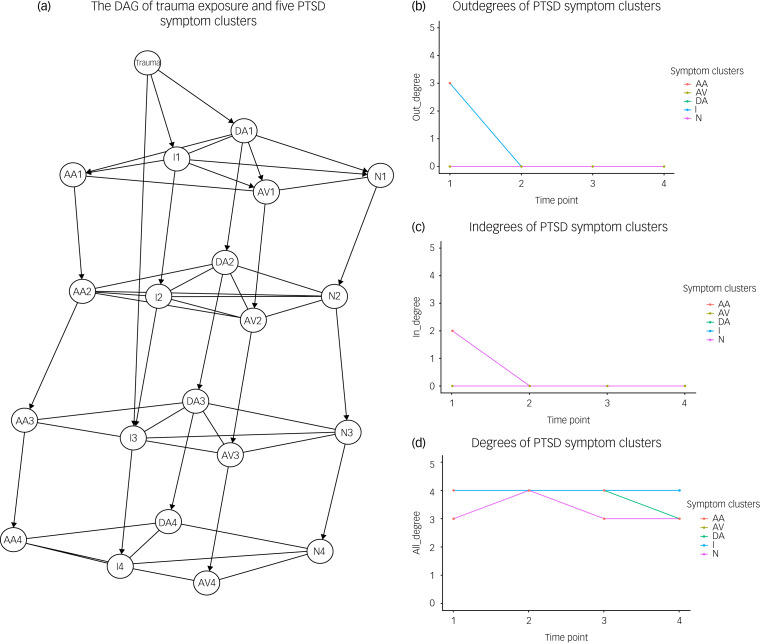
The DAG of trauma exposure and five PTSD symptom clusters and three centrality indices of nodes at four time points. (a) The DAG of trauma exposure and five PTSD symptom dimensions measured at four time points. Autoregressive connections between symptom clusters at lags >1 were suppressed visually to avoid clutter (see Supplementary Fig. 1 for the complete graph). The number after the abbreviated symptom cluster indicates the measurement time. (b) Outdegrees of PTSD symptom clusters within each of the four time points. (c) Indegrees of PTSD symptom clusters within each of the four time points. (d) Degrees of PTSD symptom clusters within each of the four time points. AA, anxious arousal; AV, avoidance; DA, dysphoric arousal; DAG, directed acyclic graph; I, intrusion; N, numbing; PTSD, post-traumatic stress disorder.

The DAG indicated that trauma exposure was only directly connected to intrusion and dysphoric arousal symptoms at time point 1, and to intrusion symptoms at time point 3 (Fig. 1(a)). Directed cross-sectional edges from intrusion and dysphoric arousal to other symptoms were only identified at time point 1. Regarding centrality of nodes, intrusion symptoms had the highest outdegree and lowest indegree at time point 1 (Figs 1(b) and 1(c)), and the highest degree at all time points (Fig. 1(d)). Auto-dependency of all PTSD symptoms was presented across all time points, whereas no cross-symptom effects across all time points were found (Fig. 1(a)). The DAG and centrality results demonstrated enough stability in bootstrap analyses (see Supplementary Figs 2 and 3). The GGMs indicated that regardless of including or not including trauma exposure, PTSD symptoms were strongly connected at all four time points (Supplementary Fig. 4).

## Discussion

To our knowledge, this is the first study in PTSD literature applying the DAG in a large longitudinal data-set, which provided a natural framework to investigate the psychopathological processes of PTSD and the role of trauma exposure. Regarding the relationship between trauma exposure and PTSD symptoms, the DAG results suggest that trauma exposure links to the network of PTSD symptoms mainly through intrusion symptoms, which act as gateway symptoms that channel the effects of trauma exposure to other PTSD symptoms, and further suggest that trauma exposure does not connect to PTSD symptoms jointly. These findings provide evidence supporting the hypothesis that trauma exposure serves as a trigger activating specific gateway symptoms, and rejecting the hypothesis that trauma exposure serves as a common cause of PTSD symptoms. Furthermore, the link between trauma exposure and intrusion symptoms was found at time points 1 and 3, and was not found at time points 2 and 4. As most of the families affected by the earthquake moved from transitional shelters to proper houses during 2012, the link found at time point 3 might be because of the significant life changes that could act as reminders of earthquake-related experiences. Thus, the finding could not provide clear evidence supporting trauma exposure as a chronic stressor in the maintenance of PTSD.

With respect to the dynamic interplay of PTSD symptoms, we found that intrusion symptoms were first activated by trauma exposure, and subsequently activated other PTSD symptoms at time point 1. This finding is generally congruent with previous longitudinal research^10^ and with the cognitive processing model of PTSD,^11^ and highlights the central role of intrusion symptoms in the development of PTSD psychopathology. Furthermore, all symptoms only predicted themselves at the next time point, and interacted undirectedly with each other at each subsequent time point. This suggests that PTSD symptoms may persistently sustain each other through (possibly reciprocal) interactions as a dynamic network in the chronicity of post-traumatic psychopathology, which is consistent with the conceptualisation of mental disorders by the network theory.^3^

The main limitations of this study included using a sample of youths exposed to a specific traumatic event, and using a dichotomous trauma exposure measure and self-report PTSD measure. Further replications with samples of youths and adults exposed to different trauma events, using clinician-administered PTSD measures and sophisticated assessments of trauma exposure that include intensity and frequency are warranted. Although limited, the current study identified the psychopathological pathway from the triggering trauma through the gateway intrusion symptoms to the onset of PTSD, informing intrusion symptoms as the potential target in developing prevention and early intervention programmes for PTSD.

## Supporting information

Chen et al. supplementary material Chen et al. supplementary material

## Data Availability

The data that support the findings of the current study are available from the corresponding author, L.W., on reasonable request. R code for this study is available from the corresponding author, L.W., on request. All materials supporting the findings of the study can be found in the Supplementary material.
