# Supplementary material for: Revealing the psychopathological pathway linking trauma to post-traumatic stress disorder: longitudinal network approach
Source: BJPsych Open. 2023 Dec 4;10(1):e2. doi: 10.1192/bjo.2023.615 (PMC10755552; doi:10.1192/bjo.2023.615)
Supplement: Chen et al. supplementary material [file S2056472423006154sup001.docx]

**Revealing the psychopathological pathway linking trauma to PTSD: a longitudinal network approach**

Chen Chen, Chengqi Cao, Ruojiao Fang, Li Wang, Denny Borsboom

**Supplementary material**

Contents:

- Measure of Trauma Exposure
- More about the DAG
- Fig 1 The Unsuppressed Directed Acyclic Graph (DAG) of trauma exposure (TE) and five PTSD symptom clusters measured at four time points.
- Fig 2 The Bootstrapped DAG (1000 times) of trauma exposure (TE) and five PTSD symptom clusters measured at four time points.
- Fig 3 Bootstrapped 95% confidence intervals (CI) for degrees, out-degrees, and in-degrees of PTSD symptom clusters within each of the four time points.
- Fig 4 Partial correlation networks of PTSD symptom clusters including and not including trauma exposure.
- Further discussion about cross-symptom effects.

**Measure of Trauma Exposure**

Earthquake-related trauma exposure was assessed at T1 by 5 questions asking whether participants experienced:

(a) traumatic death of a family member;

(b) being injured;

(c) witnessing injury of someone;

(d) witnessing buildings collapse;

(e) exposure to a corpse during the earthquake.

These five yes (1) or no (0) questions represent the most frequently reported experiences in our earlier survey of the earthquake survivors (Li et al., 2018; Liu et al., 2018; Liu et al., 2011). The sum-score of these 5 items is calculated as the extent of earthquake-related trauma exposure.

**References:**

1. Liu M, Wang L, Shi Z, Zhang Z, Zhang K, Shen J. Mental health problems among children one-year after Sichuan earthquake in China: A follow-up study. PLoS One. 2011;6(2):8–13.

2. Li G, Wang L, Cao C, Fang R, Liu P, Luo S, et al. DSM-5 posttraumatic stress symptom dimensions and health-related quality of life among Chinese earthquake survivors. Eur J Psychotraumatol [Internet]. 2018 Jan 1;9(1):1–7. Available from: https://doi.org/10.1080/20008198.2018.1468710

3. Liu L, Wang L, Cao C, Cao X, Zhu Y, Liu P, et al. Serotonin transporter 5-HTTLPR genotype is associated with intrusion and avoidance symptoms of DSM-5 posttraumatic stress disorder (PTSD) in Chinese earthquake survivors. Anxiety, Stress Coping. 2018;31(3):318–27.

4. Scutari M, Nagarajan R. Artificial Intelligence in Medicine Identifying significant edges in graphical models of molecular networks. Artif Intell Med [Internet]. 2013;57(3):207–17. Available from: http://dx.doi.org/10.1016/j.artmed.2012.12.006

**More about DAG**

**DAG in the psychopathological literature**

Directed Acyclic Graph (DAG, also known as the graphical structure of a Bayesian network), is a graphical model with unweighted directed edges between variables representing their inferenced causal relationships (i.e., their conditional independence). DAG makes casual inferences by a two-step model selection approach: first learning the graphical structure of a Bayesian network by some algorithms and then estimating the parameters of the local distribution functions conditional on the learned structure (Scutari, 2010). The estimation of DAG can be implemented in R package bnlearn (Scutari, 2010).

DAGs have been widely used in the psychopathological network literature to discover the underlying causal structure among psychopathological symptoms, such as anxiety (Heeren et al., 2021), depression(Briganti et al., 2021), and in PTSD (e.g., Bartels et al., 2019, McNally et al., 2017). Used in the field of psychopathological research, it can provide additional causal information between symptoms besides the correlation information that offered by the commonly used the Gaussian Graphical Model (GGM).

**The iamb algorithm in DAG**

Incremental Association (iamb) is a commonly used structure learning algorithm based on the Incremental Association Markov blanket (IAMB) algorithm of the same name, which is based on a two-phase selection scheme (a forward selection followed by an attempt to remove false positives). It first estimates the skeleton of the network (i.e., the undirected graph underlying the network structure) using an optimization search restricted to the Markov blanket of each node (including the parents, the children and all the nodes that share a child with that particular node), and then setting all direction of the arcs that satisfies the corresponding d-separation (direction-dependent separation, a test for conditional independence in probability distributions) statements. As a constraint-based algorithm (there are mainly two types of Bayesian network structure learning algorithms: constraint-based and score-based, see Scutari(2010) for more details), it performs better and allows for a more certain causal interpretation compared with other structure learning algorithms.

**Fig 1. The Unsuppressed Directed Acyclic Graph (DAG) of trauma exposure (TE) and five PTSD symptom clusters measured at four time points.**
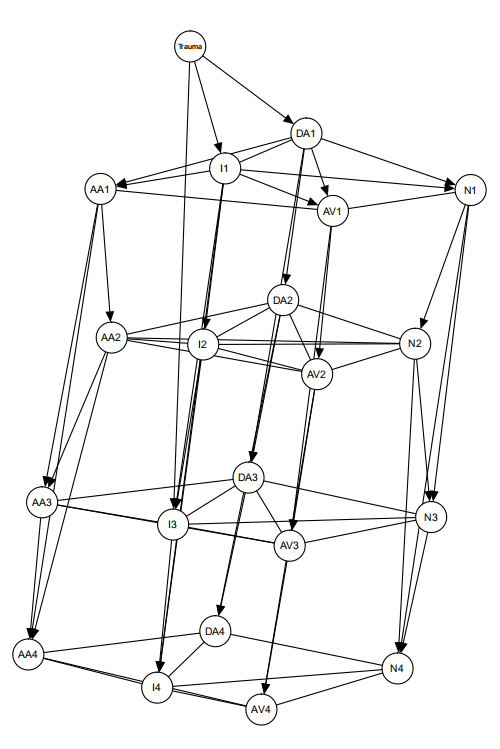


Notes: Trauma, Trauma exposure (TE); I, intrusion; AV, Avoidance; N, numbing; DA, dysphoric arousal; AA, anxious arousal. The number after the abbreviated symptom clusters indicates the measurement time. Autoregressive connections between symptom clusters at lags > 1 are suppressed visually to avoid clutter in Fig.1.

**Fig 2. The averaged bootstrapped DAG (1000 times) of trauma exposure (TE) and five PTSD symptom clusters measured at four time points.**


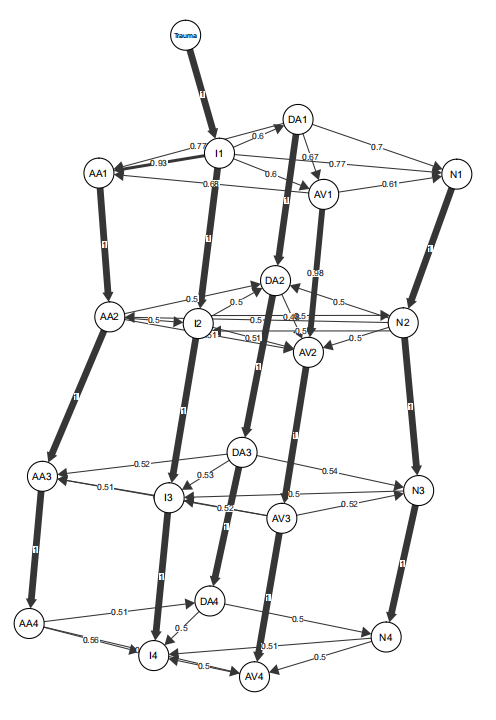


To ensure the stability of our DAG result, we bootstrapped 1000 samples and estimated one network for each sample, and computed this averaged DAG accordingly to the method suggested by Scutari and Nagarajan (2013). We first determined whether an edge is stably present or not by its strength (the frequency proportion of a given connection to appear in the bootstrapped networks), and then determined the direction of each retained edge by its odds direction (the direction of a given connection goes in over half of the bootstrapped networks). According to Scutari and Nagarajan (2013)’s recommendation, only edges that have strength > 85% are stably existent and thus presented here. Noted that there only lacked the connection from trauma to dysphoric arousal compared with Fig. 1 since this link did not appear in more than 850 DAGs of all 1000 bootstrapped networks, indicating the instability of this connection. The number on each edge indicated the proportion of this connection goes in the depicted direction in this DAG, and thus implied whether the casualty following this direction is strong or weak. Noted that except for T1, the indicated causal relationship between PTSD symptom clusters are weak (with directions between 0.5 to 0.56) at the other three time points. This is consistent with our resultant DAG where no directed edges were tested in T2, T3, and T4. In summary, this averaged bootstrapped network proved our DAG result is highly stable.

**Fig 3. Bootstrapped 95% confidence intervals (CI) for degrees, out-degrees, and in-degrees of PTSD symptom clusters within each of the four time points.**

a.


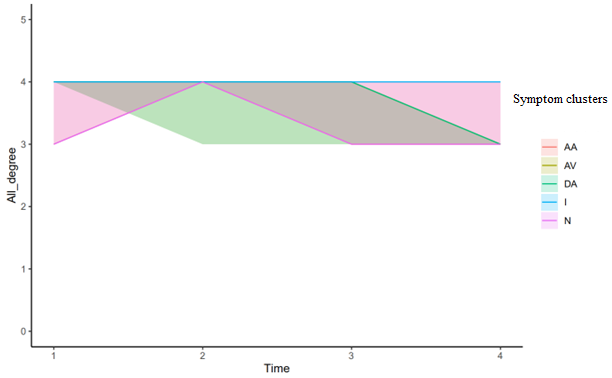


b. c.
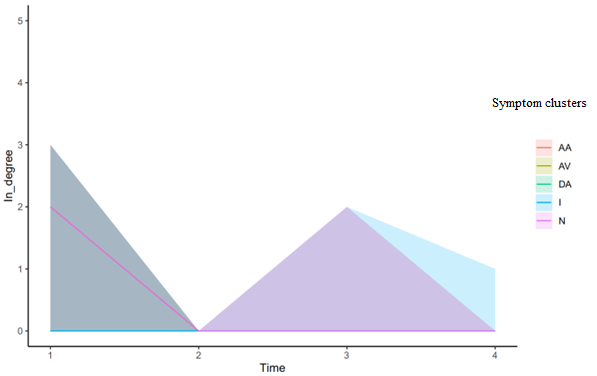
**
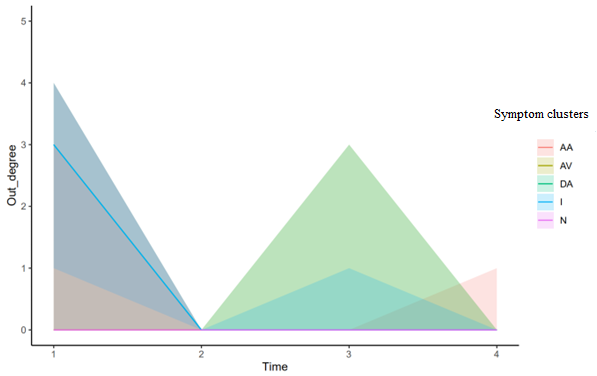
**

Notes: I, intrusion; AV, Avoidance; N, numbing; DA, dysphoric arousal; AA, anxious arousal.

The solid colourful lines indicated the degrees estimates in our sample; the correspondent shades represented the 95% CIs calculated from the 1000 bootstrapped DAGs (ranging from 0~4 since there are five nodes in total). The degrees of all five nodes in four time points fell into a 95% CI band of 3 to 4 (Fig. 3a), indicating the stably high degrees of all nodes across time. Noted that only the 95% CI of intrusion overlaps at a highest degree of 4 at all times. This means in all 1000 bootstrapped DAGs, intrusion has connections with all the other four nodes across all time. When taking directions into consideration, out-degrees (Fig. 3b) and in-degrees (Fig. 3c) of nodes demonstrated less stability. Only the out-degree of intrusion at T1 has a 95% CI that does not cover 0. These resonated with the stable connections with undetected directions in both our DAG result and the 1000 bootstrapped DAGs.

**Fig4. Partial correlation networks of PTSD symptom clusters including and not including trauma exposure.**

*
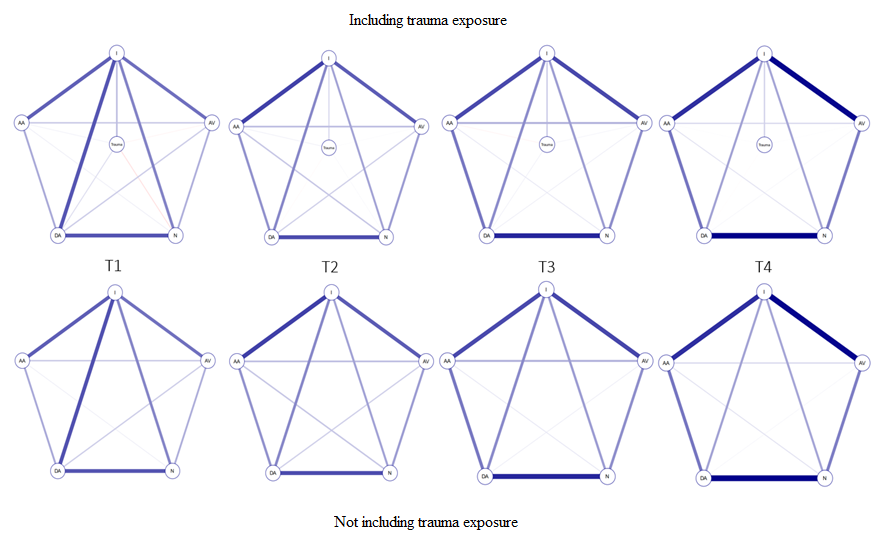
*

Notes: I, intrusion; AV, Avoidance; N, numbing; DA, dysphoric arousal; AA, anxious arousal.

**Further discussion about cross-symptom effects**

Our DAG results did not find any cross-symptom interaction between different time points. These interactions might be obscured by the relatively long assessment intervals (about a year), while these effects can play out at shorter time scales. Future research shortening the measurement lag would offer more information of cross-symptom interactions over time.
